# Futile reperfusion and predicted therapeutic benefits after successful endovascular treatment according to initial stroke severity

**DOI:** 10.1186/s12883-019-1237-2

**Published:** 2019-01-15

**Authors:** Sang-Hwa Lee, Beom Joon Kim, Moon-Ku Han, Tai Hwan Park, Kyung Bok Lee, Byung-Chul Lee, Kyung-Ho Yu, Mi Sun Oh, Jae Kwan Cha, Dae-Hyun Kim, Hyun-Wook Nah, Jun Lee, Soo Joo Lee, Jae Guk Kim, Jong-Moo Park, Kyusik Kang, Yong-Jin Cho, Keun-Sik Hong, Hong-Kyun Park, Jay Chol Choi, Joon-Tae Kim, Kangho Choi, Dong-Eog Kim, Wi-Sun Ryu, Wook-Joo Kim, Dong-Ick Shin, Minju Yeo, Sung-Il Sohn, Jeong-Ho Hong, Juneyoung Lee, Ji Sung Lee, Pooja Khatri, Hee-Joon Bae

**Affiliations:** 1 Department of Neurology, Hallym University Chuncheon Sacred Heart Hospital, Chuncheon, Korea; 2 Department of Neurology, Seoul National University Bundang Hospital, Seongnam, Korea; 3 Department of Neurology, Seoul Medical Center, Seoul, Korea; 4 Department of Neurology, Soonchunhyang University College of Medicine, Seoul, Korea; 5 Department of Neurology, Hallym University Sacred Heart Hospital, Anyang, Korea; 6 Department of Neurology, Dong-A University Hospital, Pusan, Korea; 7 Department of Neurology, Yeungnam University Medical Center, Daegu, Korea; 8 Department of Neurology, Eulji University Hospital, Eulji University School of Medicine, Daejeon, Korea; 9 Department of Neurology, Eulji General Hospital, Eulji University, Seoul, Korea; 10 Department of Neurology, Ilsan Paik Hospital, Inje University, Goyang, Korea; 11 Department of Neurology, Jeju National University, Jeju, Korea; 12 Department of Neurology, Chonnam National University Hospital, Gwangju, Korea; 13 Department of Neurology, Dongguk University Ilsan Hospital, Goyang, Korea; 14 Ulsan University Hospital, University of Ulsan College of Medicine, Ulsan, Korea; 15 Department of Neurology, Chungbuk National University College of Medicine, Cheongju, Korea; 16 Department of Neurology, Keimyung University Dongsan Medical Center, Daegu, South Korea; 17 Department of Biostatistics, Korea University College of Medicine, Seoul, Korea; 18 Clinical Research Center, Asan Medical Center, Seoul, Korea; 19 Department of Neurology, University of Cincinnati, Cincinnati, OH USA

**Keywords:** Futile reperfusion, Endovascular treatment, Stroke severity, Therapeutic benefit

## Abstract

**Background:**

Futile reperfusion (poor functional status despite successful reperfusion) was observed in up to 67% of patients enrolled in recent endovascular treatment (EVT) clinical trials. We investigated the impact of baseline stroke severity on both futile reperfusion and therapeutic benefit of successful EVT.

**Methods:**

Using a prospective multicenter stroke registry, we identified consecutive ischemic stroke patients with anterior circulation large artery occlusion, who were reperfused successfully by EVT (Thrombolysis in Cerebral Infarction grade 2b–3). The rate of futile reperfusion was assessed across the initial National Institutes of Health Stroke Scale (NIHSS) scores. The frequency of poor outcomes (modified Rankin scale [mRS] 3–6) according to NIHSS scores was compared between patients revascularized successfully by EVT and those who did not receive EVT, after standardizing for age.

**Results:**

Among 21,591 patients with ischemic stroke, 972 (4.5%) received EVT within 12 h of onset, including 440 who met study eligibility criteria. Futile reperfusion was observed in 226 of the 440 study-eligible patients (51.4%) and was associated with stroke severity: 20.9% in NIHSS scores ≤5, 34.6% in 6–10, 58.9% in 11–20, and 63.8% in > 20 (*p* < 0.001). Nonetheless, the therapeutic benefit of EVT also increased with increasing stroke severity (p for interaction < 0.001): 0.1% in NIHSS ≤5, 18.6% in 6–10, 28.7% in 11–20, and 34.3% in > 20.

**Conclusions:**

EVT is more beneficial with increasing stroke severity, although futile reperfusion also increases with higher stroke severity.

**Electronic supplementary material:**

The online version of this article (10.1186/s12883-019-1237-2) contains supplementary material, which is available to authorized users.

## Background

Futile reperfusion, defined as poor 3-month outcome (modified Rankin Scale, mRS ≥3) despite successful reperfusion (Thrombolysis in Cerebral Infarction [TICI] grade 2b to 3 reperfusion flow after endovascular treatment [EVT]) [1–3], ranged from 29 to 67% in the five pivotal EVT clinical trials published in 2015 [4–8]. Earlier studies suggested that initial stroke severity, as measured by the National Institutes of Health Stroke Scale (NIHSS), strongly predicts futile reperfusion [1, 9, 10]; in a pooled analysis, 66% of patients with initial NIHSS scores ≥20 were functionally dependent despite successful revascularization [2].

At the same time, a recent analysis [11], pooling Interventional Management of Stroke III [12] and MR CLEAN data [5], suggested that EVT might be most beneficial in severe stroke patients (NIHSS ≥20). Another pooled analysis [13] of the five 2015 EVT trials (MR CLEAN [5], ESCAPE [4], REVASCAT [8], SWIFT PRIME [7], and EXTEND IA [6]) reported that EVT was similarly effective across the whole range of NIHSS scores; the adjusted odds ratio (OR) was 2.52 (confidence intervals [CIs], 1.40–4.54) in patients with NIHSS scores ≥21, and 1.67 (CI 0.80–3.50) for NIHSS ≤10 (p for interaction = 0.45).

Futile reperfusion is a useful concept that limits study subjects to those treated successfully with EVT. It thereby leaves aside factors related to reperfusion failure related to operators, such as experience levels and diversity of techniques [14]. The clinician can then consider individual patient factors that influence outcome after reperfusion. This concept is most valuable for observational or quasi-experimental studies examining outcomes in heterogeneous, real-world settings.

Using a nationwide multicenter stroke registry database [14, 15], this study aimed to investigate whether futile reperfusion depends on initial NIHSS scores, and to estimate the therapeutic benefit of EVT across NIHSS scores by comparing those treated successfully with EVT to those not treated with EVT.

## Methods

### Study subjects

We retrospectively analyzed the Clinical Research Center for Stroke-5th division (CRCS-5) registry, a prospective, nationwide, multicenter, acute stroke database established in 2008 [14, 15]. The study group consisted of 14 academic and regional stroke centers. In the registry, we identified consecutive acute ischemic stroke patients without pre-stroke disability (mRS was 0 to 1), who had anterior circulation large artery (middle cerebral artery [MCA] including M1 and M2, or internal carotid artery [ICA]) occlusion, and who were treated successfully with EVT (TICI grade 2b or 3) within 12 h of stroke onset, between November 2009 and July 2014 (defined as *the successful EVT group)*. We evaluated determinants of futile reperfusion in this group. We also identified acute ischemic stroke patients presenting with MCA or ICA occlusions within 12 h of onset but who were not treated with EVT (defined as *the no-EVT group*). We estimated therapeutic benefits of EVT across the whole range of NIHSS scores, comparing the successful EVT and no-EVT groups, with direct standardization for age.

### Data collection and parameter definitions

From the registry database, we obtained demographics, stroke risk factors and medical history, stroke characteristics and treatments (initial NIHSS scores, pre-stroke mRS, ischemic stroke subtype according to the Trial of Org 10,172 in Acute Stroke Treatment (TOAST) criteria, with some modifications [16], preceding intravenous thrombolysis, and intervals from onset to initiating EVT, as well as laboratory data and large vessel status. Additionally, post-EVT vessel status was graded centrally, using the TICI grade, by three experienced vascular neurologists, who reviewed angiographic images (Lee J, Hong J-H, and Park H-K, kappa index = 0.85).

The primary outcome measure was the proportion of futile reperfusion, defined as a 3-month mRS score of 3 to 6 despite successful reperfusion (TICI grade 2b or 3), according to initial NIHSS scores, categorized as ≤5, 6–10, 11–20, and > 20 in the EVT group [2, 10]. The secondary outcome measure was the predicted therapeutic benefit for each NIHSS category, which was estimated as the difference between the proportion of patients with mRS scores of 3 to 6 who were treated successfully with EVT (*the successful EVT group*), and the age-standardized proportion of patients with mRS scores of 3 to 6 not treated with EVT (*the no-EVT group*).

### Statistical analysis

Summary statistics were presented as the number of subjects (percentage), for categorical variables, and as mean ± SD or median (interquartile range), for continuous variables. Group comparisons were made using Pearson’s chi-squared test for categorical variables and Student’s t-test or the Mann-Whitney U test for continuous variables, where appropriate.

Comparisons of clinical and laboratory profiles were made according to futile reperfusion status in the EVT group, and the proportion of futile reperfusion according to each initial NIHSS category was also presented. A linear trend between futile reperfusion and initial NIHSS category was examined with a chi-squared test for trend.

To estimate the therapeutic benefit of EVT according to each NIHSS category and to remove the influence of age, we standardized the age distribution of the no-EVT group (a surrogate control group) to the successful EVT group. Following this step, a simple weighted linear regression model was used to test a trend of linearity of the odds of EVT for patients with mRS scores of 0–2 compared to those with scores of mRS 3–6, according to initial NIHSS categories classified into 0–5, 6–10, 11–20, and > 20. See Additional file 1: Appendix for details.

As a post hoc analysis, we also assessed the therapeutic benefits of EVT regardless of reperfusion status. All patients who received EVT were defined as *the whole EVT group* and the therapeutic benefit of EVT according to each NIHSS category in this population was estimated, using the same standardization method and simple weighted linear regression model. Additionally, the proportions of futile reperfusion and the effect of NIHSS on futile reperfusion were obtained and compared between patients less than 80 years and 80 years or greater, in *the successful EVT group*.

Also, as a sensitivity analysis, we analyzed the proportions of futile reperfusion in patients successfully treated with EVT within 6 h of onset, and the therapeutic benefit of EVT according to each initial NIHSS category, comparing patients treated successfully with EVT within 6 h of onset and those not treated with EVT despite presenting within 6 h of onset.

To explore predictors of futile reperfusion, we analyzed *the successful EVT group* using binary logistic regression models with futile reperfusion as an outcome variable. Unadjusted and adjusted ORs and 95% CIs of potential predictors were estimated.

## Results

Among 21,591 consecutive patients, hospitalized with acute ischemic stroke over a span of 57 months, 972 (4.5%) received EVT within 12 h of stroke onset. Of those 972 patients, 533 (54.8%) were recanalized successfully with EVT (TICI grade 2b or 3), and of those 533 patients, 440 patients with anterior circulation large artery occlusion were included in *the successful EVT group* (male, 58%; age, 67.3 ± 12.3 years; onset to EVT time, 4.19 ± 1.96 h) (Additional file 2: Figure S1). In *the successful EVT group*, the overall rate of futile reperfusion (3-month mRS score of 3 to 6) was 51.4% (*n* = 226). Compared to those without futile reperfusion, those with futile reperfusion were more likely to be female, hypertensive, have higher creatinine levels, higher NIHSS scores, were less likely to be current smokers (all *p* values < 0.05; Table 1), and tended to be older (*p* = 0.08). Onset to start of EVT (i.e., arterial puncture) and the location of lesions were not associated with futile reperfusion. Symptomatic hemorrhagic transformation and neurologic progression (mostly attributable to swelling of infarcted brain or perilesional edema) were more common in patients with futile reperfusion (symptomatic hemorrhagic transformation; 8.8% vs. 2.8%, neurologic progression; 17.7% vs. 10.3%, p’-values < 0.001).

**Table 1:** Comparison of the futile reperfusion group and the no-futile reperfusion group after successful reperfusion

	Futile reperfusion (3-month mRS 3–6 with TICI grade 2b–3) *n* = 226	No-futile reperfusion (3-month mRS 0–2 with TICI grade 2b–3) *n* = 214	*P*-value
Age, mean ± SD	70.3 ± 12.1	64.0 ± 11.8	0.08^d^
Male, %	120 (53.1)	135 (63.1)	0.04^a^
TOAST			0.96^a^
LAA	51 (22.6)	50 (23.4)	
CE	131 (58.0)	121 (56.5)	
others	44 (19.5)	43 (20.1)	
Hypertension, %	151 (66.8)	122 (57.0)	0.04^a^
Diabetes mellitus, %	58 (25.7)	42 (19.6)	0.14^a^
Hyperlipidemia, %	46 (20.4)	47 (22.0)	0.73^a^
Current Smoking, %	37 (16.4)	62 (29.0)	0.002^a^
Atrial fibrillation, %	120 (53.1)	108 (50.5)	0.63^a^
NIHSS, IQR	16 (12–19)	12 (8–17)	< 0.001^c^
NIHSS ≤5	9 (4.0)	34 (15.9)	< 0.001^a^
NIHSS 6~10	28 (12.4)	53 (24.8)	
NIHSS 11~20	152 (67.3)	106 (49.5)	
NIHSS > 20	37 (16.4)	21 (9.8)	
Pre-stroke antithrombotics, %	83 (36.7)	93 (43.5)	0.17^a^
Pre-stroke statin, %	46 (20.4)	46 (21.5)	0.82^a^
SBP, mean ± SD	143.7 ± 27.3	138.4 ± 27.0	0.92^d^
Creatinine, mg/dL, mean ± SD	0.97 ± 0.48	0.92 ± 0.28	0.02^d^
Total cholesterol, mg/dL, mean ± SD	165.4 ± 39.7	166.1 ± 40.6	0.84^d^
Initial random glucose, mg/dL, mean ± SD	139.5 ± 49.2	130.0 ± 41.5	0.20^d^
Preceding IVT, %	154 (68.1)	154 (72.0)	0.41^a^
Onset to EVT start time, min, mean ± SD	257.4 ± 110.9	245.3 ± 124.0	0.18^d^
Location of occlusion, %			0.66^a^
MCA	119 (52.7)	122 (57.0)	
ICA	56 (24.8)	49 (22.9)	
MCA + ICA	51 (22.6)	43 (20.1)	
Location of lesions, %			
Right hemisphere	110 (48.7)	110 (51.4)	0.64^a^
Left hemisphere	111 (49.1)	97 (45.3)	
Both hemisphere	5 (2.2)	7 (3.3)	
Symptomatic HT	20 (8.8)	6 (2.8)	< 0.001^b^
Neurologic progression*	40 (17.7)	22 (10.3)	< 0.001^b^

* Neurologic progression was defined as 1) deterioration attributable to progressive ischemia, swelling of infarcted tissue or perilesional edema in patients who were stable neurologically during 24 h or more, 2) not attributable to stroke recurrence, symptomatic hemorrhage transformation or medical illness, and 3) an increase of total NIHSS scores 2 or more or an increase in the NIHSS subscale related to level of consciousness or motor subscale

Abbreviation: *SD* standard deviation, *mRS* modified Rankin scale, *TICI* Thrombolysis in Cerebral Infarction, *LAA* large artery atherosclerosis, *CE* cardiac embolism, *NIHSS* National Institutes of Health Stroke Scale, *IQR* interquartile range, *SBP* systolic blood pressure, *IVT* intravenous thrombolysis, *EVT* endovascular treatment, *MCA* middle cerebral artery, *ICA* internal carotid artery, *HT* hemorrhagic transformation

^a^ Calculated by chi-squared test

^b^ Calculated by Fisher exact test

^c^ Calculated by Mann-Whitney U test

^d^ Calculated by Student’s t-test

**Fig. 1:**
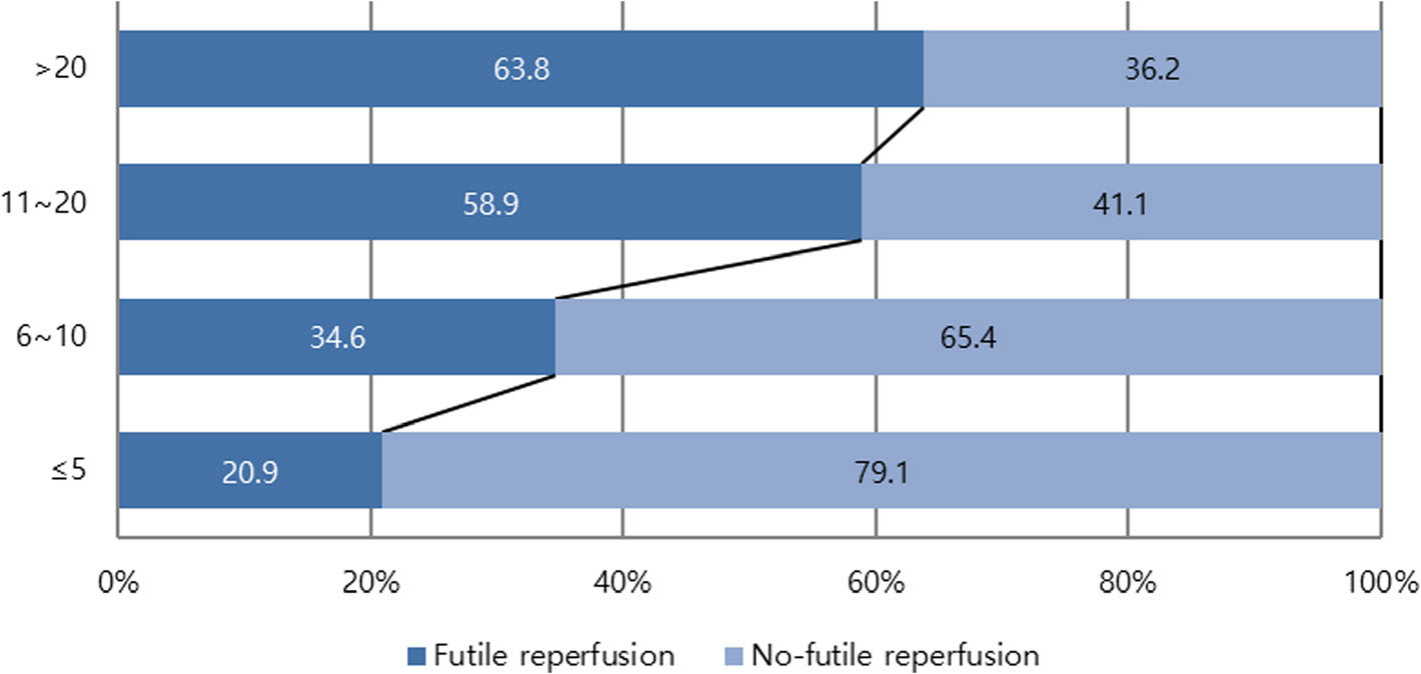
The proportion of futile reperfusion according to NIHSS in *the successful EVT group*. NIHSS, National Institutes of Health Stroke Scale; EVT, endovascular treatment

Futile reperfusion rates increased as initial NIHSS scores increased (p for trend < 0.001, Fig. 1); futile reperfusion was seen in 21% of patients with NIHSS ≤5 compared to 64% with NIHSS > 20.

**Fig. 2:**
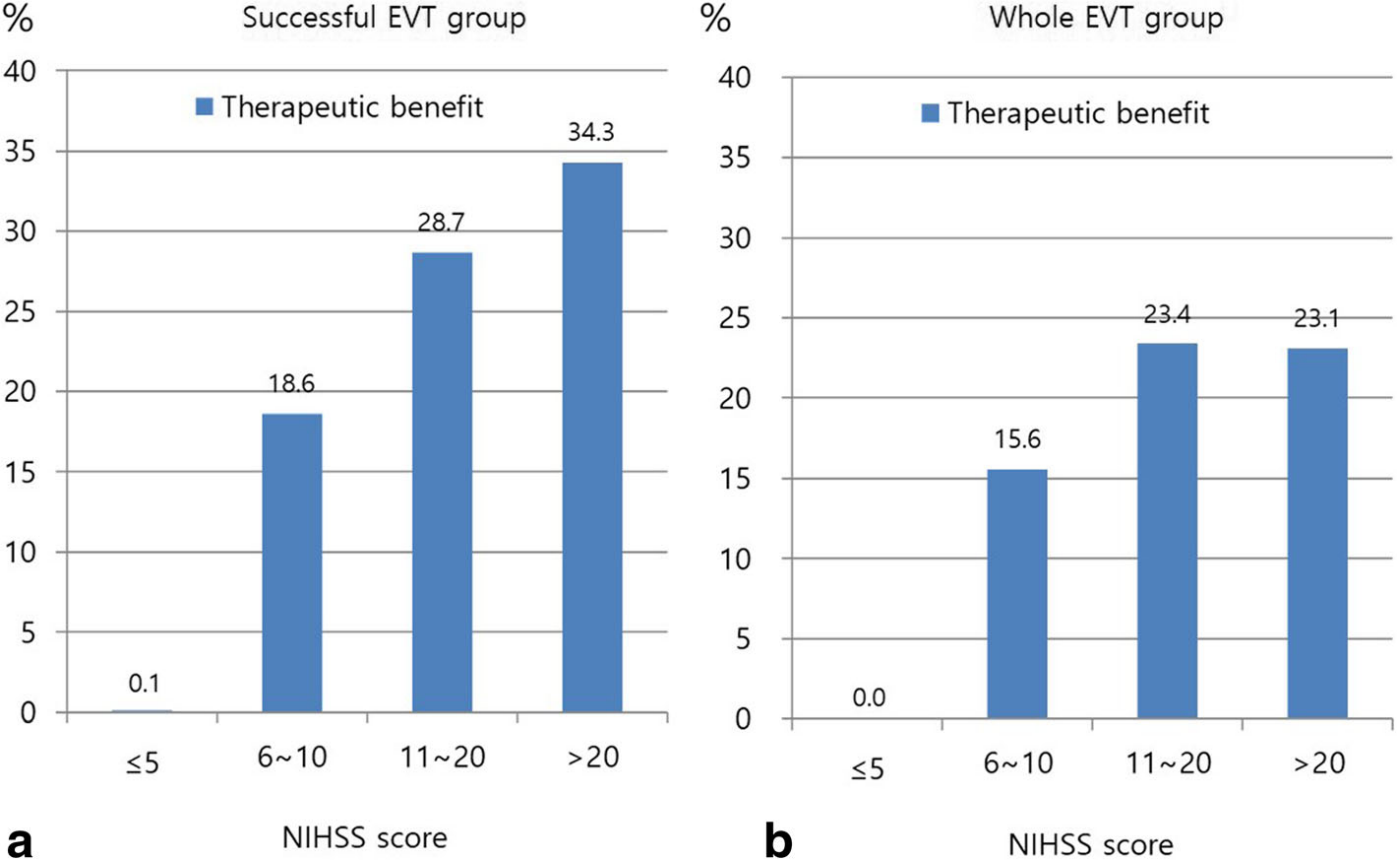
The predicted therapeutic benefits according to each of 4 initial NIHSS categories. The therapeutic benefits according to NIHSS of the *successful EVT group* (including only TICI grade 2b to 3, **a**) and of the *whole EVT group* (regardless of reperfusion status, **b**) show increasing patterns as increasing NIHSS. EVT, endovascular treatment; NIHSS, National Institutes of Health Stroke Scale

However, the predicted therapeutic benefits of EVT also increased as initial NIHSS scores increased (Fig. 2a; *p* = 0.04 for the linear trend test of ORs). Patients with NIHSS scores ≤5 experienced the lowest likelihood of therapeutic benefit from EVT (0.1%), while those with NIHSS scores > 20 experienced the highest likelihood of benefit (34%). The methods for age-specific direct standardization are detailed in Additional file 3: Table S1.

Multivariable analysis showed that, among patients with successful reperfusion, older age and higher initial NIHSS scores increased the chance of futility, while pre-stroke antithrombotic medications decreased it (Table 2). Time from onset of stroke to start of EVT was not associated with futile reperfusion. Time to reperfusion data were not available.

**Table 2:** Predictors of futile reperfusion: results of multivariable analysis

	Unadjusted OR	95% CI	Adjusted OR^a^	95% CI
Age	1.05	1.03–1.06	1.04	1.02–1.06
Male	0.66	0.45–0.97	0.67	0.43–1.05
NIHSS per 1 point	1.11	1.07–1.15	1.12	1.08–1.17
Hypertension	1.52	1.03–2.24	1.28	0.81–2.04
Diabetes Mellitus	1.41	0.90–2.22	1.10	0.61–1.96
Pre-stroke antithrombotics	0.76	0.52–1.11	0.53	0.32–0.79
Creatinine	1.42	0.85–2.38	1.11	0.60–2.07
Initial random glucose per 10 mg/dL increase	1.05	1.00–1.09	1.05	0.99–1.10
Onset to EVT start time per 10 min	1.01	0.99–1.03	1.01	0.99–1.04
Preceding IVT	0.83	0.55–1.25	0.75	0.45–1.26

Abbreviation: *OR* odds ratio, *CI* confidence interval, *NIHSS* National Institutes of Health Stroke Scale, *EVT* endovascular treatment, *IVT* intravenous thrombolysis

^a^ Adjusted for age, male, NIHSS, hypertension, diabetes mellitus, pre-stroke antithrombotic, creatinine, initial random glucose, onset to ET start time, preceding IVT

As a post hoc analysis, of those 972 patients who received EVT, 784 (80.7%) with anterior circulation large artery occlusion were analyzed regardless of reperfusion status as *the whole EVT group* (Additional file 2: Figure S1). This post hoc analysis revealed that the predicted therapeutic benefits of EVT also increased with the increase of the initial NIHSS score (Fig. 2b). The OR for favorable outcomes significantly increased as initial stroke severity increased (*p* = 0.02 for the linear trend test of ORs). The methods for age-specific direct standardization in this group are also detailed in Additional file 4: Table S2. An additional post hoc analysis with age stratification in *the successful EVT group* showed that futile reperfusion was more common in patients aged 80 years or more, compared to patients aged less than 80 years (22.1% versus 5.1%, *p* < 0.001, Additional file 5: Figure S2) but the impact of NIHSS on futile recanalization seemed to be similar between both age groups, although the increase of futile recanalization rates with increasing NIHSS scores lost statistical significance in those patients aged 80 or more (Additional file 6: Figure S3).

Sensitivity analyses of patients treated with EVT within 6 h of onset showed that the futile reperfusion rate also increased with increasing NIHSS scores (p for trend < 0.001, Additional file 7: Figure S4). In fact, patients with the lowest NIHSS scores (NIHSS≤5) showed negative therapeutic benefits of EVT (Additional file 8: Figure S5). The predicted therapeutic benefits of EVT also showed a trend towards increasing as NIHSS scores increased (*p* = 0.051 for the linear trend test of ORs).

## Discussion

Our study results suggest that the therapeutic benefits of EVT increase with an increase in stroke severity, despite a parallel increase in futile reperfusion rates, in acute stroke patients with anterior circulation artery occlusion.

Our real-world study showed that about half (51%) of patients treated successfully with EVT remain in a poor functional status. Our results are similar to those of prior studies. A multicenter EVT registry (the ENDOSTROKE study) reported a futile reperfusion rate of 59% [10]. Recent EVT trials reported futile reperfusion rates (3-month mRS 3–6) ranging from 29 to 67% [4–8, 12, 17]. This persistent high proportion of futile reperfusion mandates further research.

Our demonstration of concurrently increasing futile reperfusion and therapeutic benefits, with increasing initial stroke severity, is novel. Because most previous EVT trials [4–8, 12] included EVT-eligible patients with moderate to severe neurological deficits only (the range of median NIHSS scores was 15–18), an assessment of the absolute therapeutic benefits across the full range of initial stroke severities may not have been adequately powered. The reproduction of the study results when analyzing *the whole EVT group* rather than *the successful EVT group*, and also when limiting study subjects to those treated within 6 h of onset, demonstrates the robustness of our findings.

Notably, our study showed the lowest benefit of EVT in patients with the lowest NIHSS scores (NIHSS ≤5) and a futile reperfusion rate of 21% in this NIHSS category. The current American Heart Association guidelines are ambiguous regarding the role of EVT in acute stroke patients with intracranial ICA or MCA occlusions and initial NIHSS < 6; they state that EVT “may be reasonable” when treatment can be initiated within 6 h of onset (Class 2b, Level of Evidence B-R) [18]. As shown in Additional file 2: Table S1, the proportion of patients with 3-month mRS scores of 3 to 6, who had initial NIHSS scores ≤5, was not significantly different between *the successful EVT group* and the *no-EVT group* (20.9% versus 24.0%, respectively; *p* = 0.65). Therefore, our primary study results suggest that there are no apparent therapeutic benefits of EVT in patients with mild neurologic deficits. These findings correspond to the results of the subgroup analysis in the recent meta-analysis of the five pivotal EVT trials [13], indicating a non-significant OR of 1.67 (95% CI, 0.80 to 3.50) in patients with NIHSS ≤10. While the recent meta-analysis did not demonstrate the linearity of those ORs, it should be noted that only 14 patients, out of a total of 1287 patients, were enrolled with NIHSS ≤5 [13]. Not surprisingly, our study also showed that clinicians typically did not initiate EVT in mild stroke patients (Additional file 9: Table S3); only 5.6% were treated with EVT among acute ischemic patients with major anterior circulation occlusion and NIHSS≤5. A randomized trial testing EVT is needed in this patient population with NIHSS ≤5.

The positive associations of age and NIHSS score with futile reperfusion are concordant with the results of earlier studies [9, 10]. However, the negative association of pre-stroke antithrombotics with futile reperfusion is not consistent with a prior subgroup analysis of MR CLEAN [19]. Prior studies of antithrombotics in patients treated with intravenous (IV) thrombolysis are controversial; a small-sized observational study reported better recanalization rates and outcomes associated with pre-stroke antithrombotics [20], whereas the recent meta-analysis showed contradictory results [21].

Interestingly, onset to EVT start time was not associated with futile reperfusion. Previous studies suggested that the effectiveness of EVT for acute ischemic stroke is critically time dependent [2, 22–24]. This discrepancy might be explained, at least in part, by the use of EVT start time rather than reperfusion time in our study.

Our study has several limitations. First, the fact that this prospective multicenter registry database consisted of Korean patients treated at primarily university hospitals or other high-level regional stroke centers threatens the study’s generalizability. However, the consistency of our findings with prior studies is reassuring [11, 13]. Second, the methods of recanalization therapy (e.g., IV thrombolysis prior to EVT and tissue plasminogen activator [tPA] dose) were heterogeneous, challenging the study’s validity. However, adjusting for tPA dose in multivariable models did not change the results. Third, we did not collect the Alberta Stroke Program Early Computed Tomography (ASPECT) Scores [4] as well as pre-EVT brain images, although image parameters can be useful determinants of futile reperfusion. Forth, *the no-EVT group* was used as a surrogate control group through age-specific direct standardization in this retrospective analysis, and this may not sufficiently remove baseline imbalances between those offered EVT and those who were not (i.e., confounding by indication). Reasons (e.g., ASPECT Score [4], perfusion imaging [6], and DWI/perfusion mismatch [25], etc.) for not performing the EVT in *the no-EVT group* were not available in our registry database. Comparisons between patients treated with EVT (*the whole EVT group*) and not treated with EVT (*the no-EVT group*) (Additional file 10: Table S4) suggest that delay of onset to arrival and mild neurological deficits may be partially responsible, again implying caution regarding generalizing the study results. However, our intention was not to evaluate the effectiveness or efficacy of EVT, but to assess therapeutic gains according to initial stroke severity by directly comparing patients reperfused successfully with EVT and those who were not treated with EVT, from real-world practice. By removing patients who failed to be reperfused with EVT, we tried to reduce the biases related to selection of EVT candidates and heterogeneity of EVT techniques. Fifth, stroke subtype was not considered in this study, which affects stroke outcomes and might be associated with EVT decision. We could not adjust for stroke subtype in analysis of therapeutic benefits, like other recent EVT trials [4, 5, 7, 8], because information on stroke subtype is unavailable or uncertain before EVT decision in clinical practice and its potential influence might be attenuated by stratification by NIHSS scores. (Additional file 11: Table S5). Sixth, there were no general protocols for EVT across the participating centers. However, EVT was decided and performed at the discretion of expert stroke physicians in participating centers in accordance with the ASA/AHA and Korean stroke guidelines [18, 26, 27]. Lastly, as described above, we were unable to obtain data regarding onset to reperfusion times or specific EVT devices used, and these factors could critically affect clinical outcomes. An important thing that should be noted for this study, the concept of “futile reperfusion” [1] is not referring to the overall futility of treatment. For example, while more patients with older age and higher NIHSS will have poor outcomes with reperfusion (i.e, futile reperfusion), this group of patients will have overall better outcomes with EVT treatment; this well established by randomized trial results. Our findings, by showing worse outcomes in patients with futile reperfusion, as compared to those with no reperfusion attempted, reinforce the findings of randomized trials with real-world data.

## Conclusion

Our study suggests that both futility of EVT and therapeutic gains of EVT increase as stroke severity increases. By confining this analysis to those patients who were successfully reperfused, we provide clinicians with considerations regarding predicted outcomes with EVT. While individual trials and pooled analyses have already shown the benefit of EVT in subgroups with older age and higher NIHSS, we believe that this demonstration is helpful for the clinician to understand the likely benefit of EVT in real-world practice. However, despite the robustness of our findings, clinicians should be cautious in making treatment decisions based on this retrospective analysis alone. Our data, along with empiric evidence from recent randomized trials, suggest that EVT is most beneficial for acute stroke patients with moderate to severe neurological deficits, and further study is needed in patients with mild deficits.

## Additional files

Additional file 1: Appendix. (DOCX 14 kb)

Additional file 2: **Figure S1**. Flow chart of study (DOCX 57 kb)

Additional file 3: **Table S1**. Summary of direct standardization process to estimate therapeutic benefits of the successful EVT (including only TICI grade 2b to 3) according to NIHSS category. (DOCX 20 kb)

Additional file 4: **Table S2**. Summary of direct standardization process to estimate therapeutic benefits of *the whole EVT* (regardless of reperfusion status) according to NIHSS category. (DOCX 19 kb)

Additional file 5: **Figure S2**. The proportion of futile reperfusion according to age (< 80 and ≥ 80) in *the successful EVT group. (DOCX 39 kb)*

Additional file 6: **Figure S3**. The proportions of futile recanalization according to NIHSS scores in aged < 80 and ≥ 80 in *the successful EVT group (DOCX 63 kb)*

Additional file 7: **Figure S4**. The proportion of futile reperfusion according to each initial NIHSS category in *the successful EVT group* as a sensitivity analysis of EVT-treated patients within 6 h of onset. (DOCX 26 kb)

Additional file 8: **Figure S5**. The predicted therapeutic benefits of *the successful EVT* group (including only TICI grade 2b to 3) according to each of 4 initial NIHSS categories as a sensitivity analysis of EVT-treated patients within 6 h of onset. (DOCX 31 kb)

Additional file 9: **Table S3**. Proportions of EVT according to NIHSS category in acute ischemic stroke patients (*n* = 3117) who were treated with EVT or hospitalized within 12 h of onset and had causative ICA or MCA occlusion. (DOCX 15 kb)

Additional file 10: **Table S4**. Comparison of *the Whole EVT group* (regardless of reperfusion status) and *the no-EVT group (DOCX 20 kb)*

Additional file 11: **Table S5**. Distribution of stroke subtype by stratification of stroke severity in the Whole EVT group and no-EVT group. (DOCX 15 kb)

## Abbreviations

ASPECT: Alberta Stroke Program Early Computed Tomography
CE: Cardiac embolism
CRCS-5: Clinical Research Center for Stroke-5th division
DWI: Diffusion weighted imaging
EVT: Endovascular treatment
ICA: Internal carotid artery
IV: Intravenous
LAA: Large artery atherosclerosis
MCA: Middle cerebral artery
mRS: Modified rankin scale
NIHSS: National Institutes of Health Stroke Scale
TICI: Thrombolysis in Cerebral Infarction
TOAST: Trial of Org 10,172 in Acute Stroke Treatment
tPA: Tissue plasminogen activator
