# Supplementary material for: Futile reperfusion and predicted therapeutic benefits after successful endovascular treatment according to initial stroke severity
Source: BMC Neurol. 2019 Jan 15;19:11. doi: 10.1186/s12883-019-1237-2 (PMC6332890; doi:10.1186/s12883-019-1237-2)
Supplement: Supplementary file 1 — Appendix. (DOCX 14 kb) [file 12883_2019_1237_MOESM1_ESM.docx]

Additional file 1: Appendix

Detailed statistical methods for age-specific standardization and simple weighted linear regression model as below;

First, we calculated the age-specific proportion of 3-month mRS 3–6 for each age interval (<65, 65~74, >74 years) by each NIHSS category in both the *successful EVT* and *no-EVT groups*. Second, the age- and NIHSS-specific 3-month mRS 3-6 proportion in *the no-EVT group* was multiplied by the proportion of each age interval in each NIHSS category of *the successful EVT group*, respectively, and was summed up in each NIHSS category to obtain an age-standardized proportion of 3-month mRS 3-6 according to the initial NIHSS category. Then, by subtracting the proportion of 3-month mRS 3-6 in each NIHSS category of *the successful EVT group* from this age-standardized proportion, we estimated the predicted therapeutic benefits of EVT according to the initial NIHSS category.

In this analysis, a natural logarithm of the OR [i.e., ln(OR)] for each of NHISS category was used as a dependent variable by noting that the ln(OR) followed normal distribution asymptotically, while midpoints of initial NHISS categories (namely, 2.5, .8.0, 15.5, and 22.5, respectively) was used as an independent variable. As a weight, an inverse of the standard error of the ln(OR) was used
