# Supplementary material for: Futile reperfusion and predicted therapeutic benefits after successful endovascular treatment according to initial stroke severity
Source: BMC Neurol. 2019 Jan 15;19:11. doi: 10.1186/s12883-019-1237-2 (PMC6332890; doi:10.1186/s12883-019-1237-2)
Supplement: Supplementary file 2 — Figure S1. Flow chart of study (DOCX 57 kb) [file 12883_2019_1237_MOESM2_ESM.docx]

Additional file 2 Figure S1. Flow chart of study


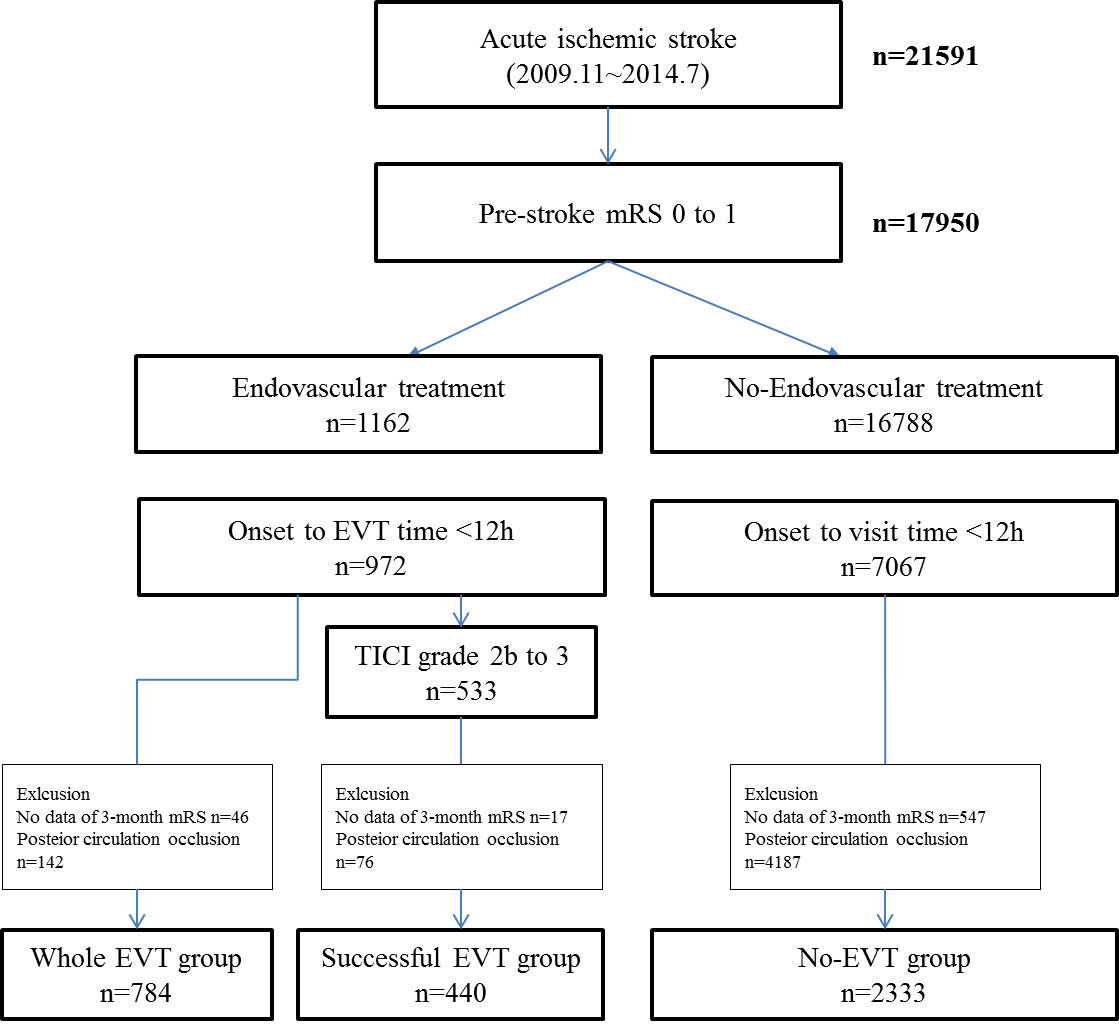


mRS indicates modified Rankin Scale; EVT, endovascular treatment; TICI, thrombolysis in Cerebral infarction
