# Supplementary material for: Futile reperfusion and predicted therapeutic benefits after successful endovascular treatment according to initial stroke severity
Source: BMC Neurol. 2019 Jan 15;19:11. doi: 10.1186/s12883-019-1237-2 (PMC6332890; doi:10.1186/s12883-019-1237-2)
Supplement: Supplementary file 3 — Table S1. Summary of direct standardization process to estimate therapeutic benefits of the successful EVT (including only TICI grade 2b to 3) according to NIHSS category. (DOCX 20 kb) [file 12883_2019_1237_MOESM3_ESM.docx]

Additional file 3: Table S1. Summary of direct standardization process to estimate therapeutic benefits of the successful EVT (including only TICI grade 2b to 3) according to NIHSS category

| 1. Proportion of 3-month mRS 3-6 by each age and NIHSS category in *the no-EVT group* | | | | | | | | | | |
| --- | --- | --- | --- | --- | --- | --- | --- | --- | --- | --- |
| NIHSS | Age <65,  mRS 3-6, n | Age <65,  mRS 0-6, n | Age<65,  mRS 3-6, % | Age 65~74,  mRS 3-6, n | Age 65~74,  mRS 0-6, n | Age 65~74,  mRS 3-6, % | Age ≥75,  mRS 3-6, n | Age ≥75,  mRS 0-6, n | Age ≥75,  mRS 3-6, % | Avg |
| ≤5 | 53 | 390 | 13.6^a^ | 86 | 390 | 22.1 | 143 | 397 | 36.0 | 24.0 |
| 6~10 | 42 | 101 | 41.6 | 64 | 122 | 52.5 | 135 | 187 | 72.2 | 58.8 |
| 11~20 | 125 | 149 | 83.9 | 141 | 161 | 87.6 | 261 | 283 | 92.2 | 88.9 |
| >20 | 20 | 20 | 100.0 | 34 | 35 | 97.1 | 96 | 98 | 98.0 | 98.0 |
| 2. Proportion of 3-month mRS 3-6 by each age and NIHSS category in *the successful EVT group* | | | | | | | | | | |
| NIHSS | Age <65,  mRS 3-6, n | Age <65,  mRS 0-6, n | Age <65,  mRS 3-6, % | Age 65~74,  mRS 3-6, n | Age 65~74,  mRS 0-6, n | Age 65~74,  mRS 3-6, % | Age ≥75,  mRS 3-6, n | Age ≥75,  mRS 0-6, n | Age ≥75,  mRS 3-6, % | Avg^1^ |
| ≤5 | 1 | 17^b^ | 5.9 | 6 | 19 | 31.6 | 2 | 7 | 28.6 | 20.9 |
| 6~10 | 12 | 33 | 36.4 | 7 | 27 | 25.9 | 9 | 21 | 42.9 | 34.6 |
| 11~20 | 39 | 96 | 40.6 | 52 | 83 | 62.7 | 61 | 79 | 73.5 | 58.9 |
| >20 | 5 | 12 | 41.7 | 14 | 20 | 70.0 | 18 | 26 | 69.2 | 63.8 |
| 3. Standardized proportion of 3-month mRS 3-6 in *the no-EVT* group using age-specific direct standardization | | | | | | | | | | |
| NIHSS | Age <65,  mRS 3-6, n | Age <65,  mRS 0-6, n | Age <65,  mRS 3-6, % | Age 65~74,  mRS 3-6, n | Age 65~74,  mRS 0-6, n | Age 65~74,  mRS 3-6, % | Age ≥75,  mRS 3-6, n | Age ≥75,  mRS 0-6, n | Age ≥75,  mRS 3-6, % | Avg^2^ |
| ≤5 | 2.3^c^ | 17 | 13.6 | 4.2 | 19 | 22.1 | 2.5 | 7 | 36.0 | 21.0 |
| 6~10 | 13.7 | 33 | 41.6 | 14.2 | 27 | 52.5 | 15.2 | 21 | 72.2 | 53.1 |
| 11~20 | 80.5 | 96 | 83.9 | 72.7 | 83 | 87.6 | 72.9 | 79 | 92.2 | 87.6 |
| >20 | 12.0 | 12 | 100.0 | 19.4 | 20 | 97.1 | 25.5 | 26 | 98.0 | 98.1 |
| 4.Predicted therapeutic benefits according to each NIHSS category (Avg^2^ minus Avg^1^) | | | | | | | | | | |
| NIHSS | % | | | |  |  |  |  |  |  |
| ≤5 | 0.1 | | | |  |  |  |  |  |  |
| 6~10 | 18.6 | | | |  |  |  |  |  |  |
| 11~20 | 28.7 | | | |  |  |  |  |  |  |
| >20 | 34.3 | | | |  |  |  |  |  |  |

The standardized proportion of 3-month mRS 3-6 in *the no-EVT group* was calculated by multiplying the crude proportion of those of each age interval in the no-EVT group by the total population of each age interval and categorized NIHSS in *the successful EVT group*. For example, the standardized proportion of age <65 in NIHSS ≤5 (c) was obtained by multiplying the proportion of 3-month 3-6 of the same age interval and NIHSS in *the no-EVT group* (a) by the population of those of the same age and NIHSS in *the successful EVT group* (b) and dividing by 100 (c=a * b/100). Furthermore, the average of the standardized proportion of 3-month mRS 3-6 according to the categorized NIHSS (Avg^2^) was calculated by dividing the sum of the standardized proportion of 3-month 3-6 in each NIHSS category by the total population in the same NIHSS category. Finally, the predicted therapeutic benefits according to NIHSS scores were equal to Avg^2^ minus Avg^1^.
