# Supplementary material for: Futile reperfusion and predicted therapeutic benefits after successful endovascular treatment according to initial stroke severity
Source: BMC Neurol. 2019 Jan 15;19:11. doi: 10.1186/s12883-019-1237-2 (PMC6332890; doi:10.1186/s12883-019-1237-2)
Supplement: Supplementary file 4 — Table S2. Summary of direct standardization process to estimate therapeutic benefits of the whole EVT (regardless of reperfusion status) according to NIHSS category. (DOCX 19 kb) [file 12883_2019_1237_MOESM4_ESM.docx]

Additional file 4: Table S2. Summary of direct standardization process to estimate therapeutic benefits of *the whole EVT* (regardless of reperfusion status) according to NIHSS category

| 1. Proportion of 3-month mRS 3-6 by each age and NIHSS category in *the no-EVT group* | | | | | | | | | | |
| --- | --- | --- | --- | --- | --- | --- | --- | --- | --- | --- |
| NIHSS | Age <65,  mRS 3-6, n | Age <65,  mRS 0-6, n | Age<65,  mRS 3-6, % | Age 65~74,  mRS 3-6, n | Age 65~74,  mRS 0-6, n | Age 65~74,  mRS 3-6, % | Age ≥75,  mRS 3-6, n | Age ≥75,  mRS 0-6, n | Age ≥75,  mRS 3-6, % | Avg |
| ≤5 | 53 | 390 | 13.6^a^ | 86 | 390 | 22.1 | 143 | 397 | 36.0 | 24.0 |
| 6~10 | 42 | 101 | 41.6 | 64 | 122 | 52.5 | 135 | 187 | 72.2 | 58.8 |
| 11~20 | 125 | 149 | 83.9 | 141 | 161 | 87.6 | 261 | 283 | 92.2 | 88.9 |
| >20 | 20 | 20 | 100.0 | 34 | 35 | 97.1 | 96 | 98 | 98.0 | 98.0 |
| 2. Proportion of 3-month mRS 3-6 by each age and NIHSS category in *the whole EVT group* | | | | | | | | | | |
| NIHSS | Age <65,  mRS 3-6, n | Age <65,  mRS 0-6, n | Age <65,  mRS 3-6, % | Age 65~74,  mRS 3-6, n | Age 65~74,  mRS 0-6, n | Age 65~74,  mRS 3-6, % | Age ≥75,  mRS 3-6, n | Age ≥75,  mRS 0-6, n | Age ≥75,  mRS 3-6, % | Avg^1^ |
| ≤5 | 3 | 27^b^ | 11.1 | 9 | 30 | 30.0 | 3 | 13 | 23.1 | 21.4 |
| 6~10 | 23 | 54 | 42.6 | 12 | 45 | 26.7 | 16 | 36 | 44.4 | 37.8 |
| 11~20 | 76 | 157 | 48.4 | 119 | 173 | 68.8 | 111 | 145 | 64.2 | 64.4 |
| >20 | 10 | 20 | 50.0 | 24 | 31 | 77.4 | 44 | 53 | 83.0 | 75.0 |
| 3. Standardized proportion of 3-month mRS 3-6 in *the no-EVT* group using age-specific direct standardization | | | | | | | | | | |
| NIHSS | Age <65,  mRS 3-6, n | Age <65,  mRS 0-6, n | Age <65,  mRS 3-6, % | Age 65~74,  mRS 3-6, n | Age 65~74,  mRS 0-6, n | Age 65~74,  mRS 3-6, % | Age ≥75,  mRS 3-6, n | Age ≥75,  mRS 0-6, n | Age ≥75,  mRS 3-6, % | Avg^2^ |
| ≤5 | 3.7^c^ | 27 | 13.6 | 6.6 | 30 | 22.1 | 4.7 | 13 | 36.0 | 21.4 |
| 6~10 | 22.5 | 54 | 41.6 | 23.6 | 45 | 52.5 | 26.0 | 36 | 72.2 | 53.1 |
| 11~20 | 131.7 | 157 | 83.9 | 151.5 | 173 | 87.6 | 133.7 | 145 | 87.8 | 87.8 |
| >20 | 20.0 | 20 | 100.0 | 30.1 | 31 | 97.1 | 51.9 | 53 | 98.1 | 98.1 |
| 4.Predicted therapeutic benefits according to each NIHSS category (Avg^2^ minus Avg^1^) | | | | | | | | | | |
| NIHSS | % | | | |  |  |  |  |  |  |
| ≤5 | 0.0 | | | |  |  |  |  |  |  |
| 6~10 | 15.6 | | | |  |  |  |  |  |  |
| 11~20 | 23.4 | | | |  |  |  |  |  |  |
| >20 | 23.1 | | | |  |  |  |  |  |  |

The standardized proportion of 3-month mRS 3-6 in *the no-EVT group* was calculated by multiplying the crude proportion of those of each age interval in *the no-EVT group* by the total population of each age interval and categorized NIHSS in *the whole EVT group*. For example, the standardized proportion of age <65 in NIHSS ≤5 (c) was obtained by multiplying the proportion of 3-month 3-6 of the same age interval and NIHSS in *the no-EVT group* (a) by the population of those of the same age and NIHSS in *the whole EVT group* (b) and dividing by 100 (c=a * b/100). Furthermore, the average of the standardized proportion of 3-month mRS 3-6 according to the categorized NIHSS (Avg^2^) was calculated by dividing the sum of the standardized proportion of 3-month 3-6 in each NIHSS category by the total population in the same NIHSS category. Finally, the predicted therapeutic benefits according to NIHSS scores were equal to Avg^2^ minus Avg^1^.
