# Supplementary material for: Futile reperfusion and predicted therapeutic benefits after successful endovascular treatment according to initial stroke severity
Source: BMC Neurol. 2019 Jan 15;19:11. doi: 10.1186/s12883-019-1237-2 (PMC6332890; doi:10.1186/s12883-019-1237-2)

Additional file 5: Figure S2. The proportion of futile reperfusion according to age (<80 and ≥80) in *the successful EVT group*


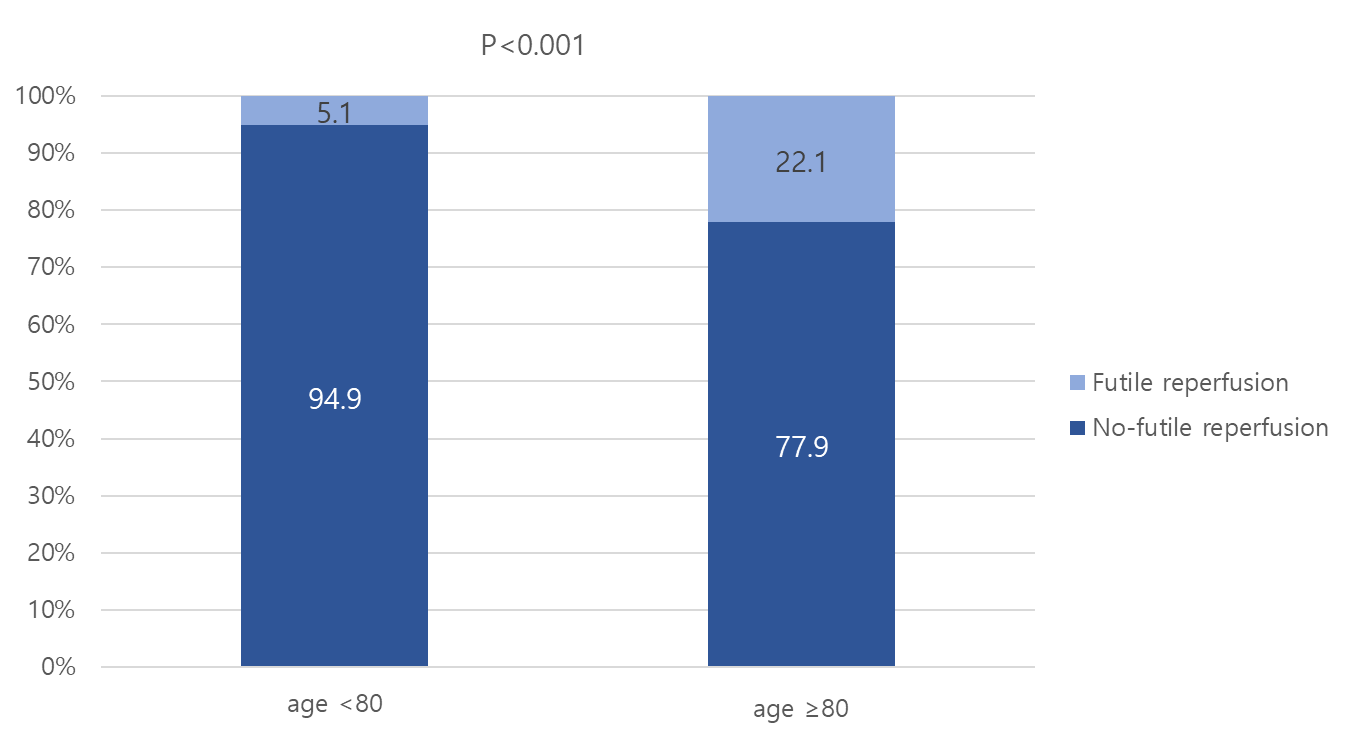

Supplement: Supplementary file 5 — Figure S2. The proportion of futile reperfusion according to age (< 80 and ≥ 80) in the successful EVT group. (DOCX 39 kb) [file 12883_2019_1237_MOESM5_ESM.docx]
