# Supplementary material for: Futile reperfusion and predicted therapeutic benefits after successful endovascular treatment according to initial stroke severity
Source: BMC Neurol. 2019 Jan 15;19:11. doi: 10.1186/s12883-019-1237-2 (PMC6332890; doi:10.1186/s12883-019-1237-2)

Additional file 6: Figure S3. The proportions of futile recanalization according to NIHSS scores in aged <80 and ≥80 in *the successful EVT group*


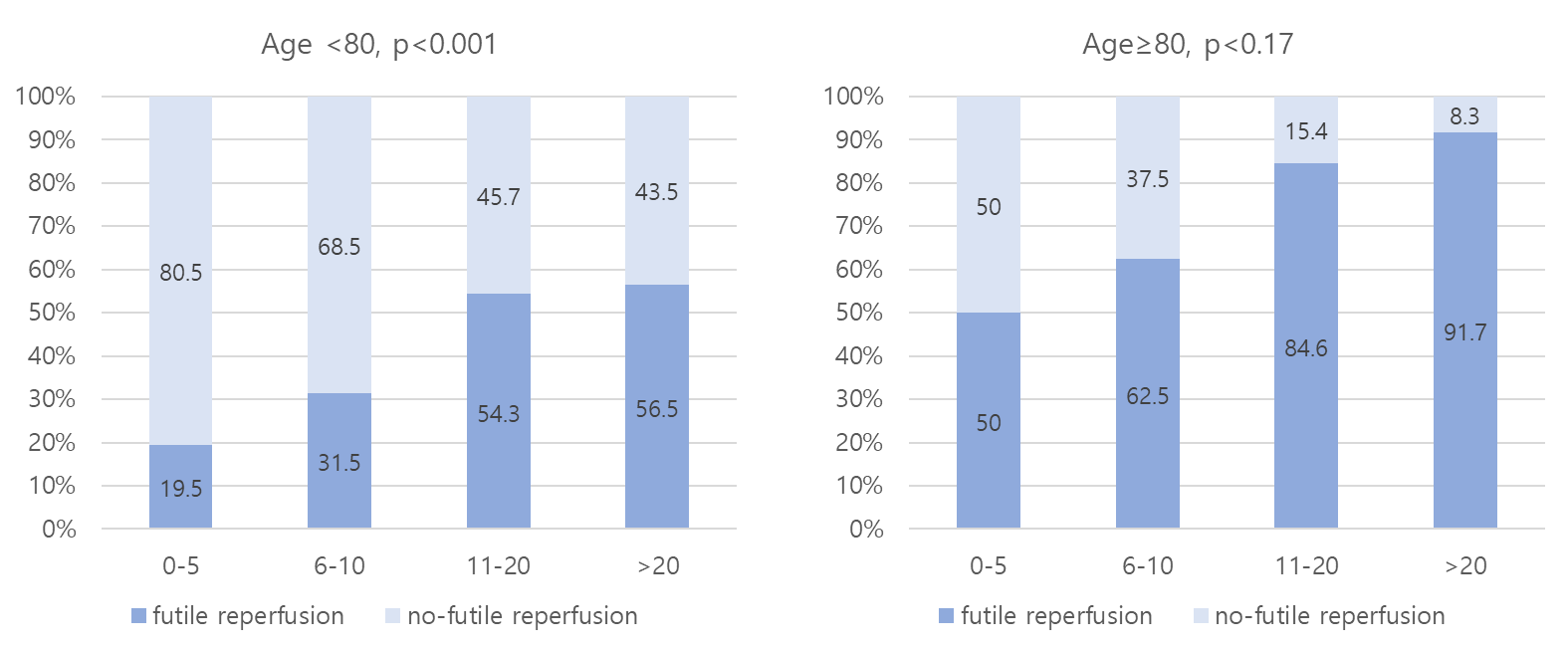

Supplement: Supplementary file 6 — Figure S3. The proportions of futile recanalization according to NIHSS scores in aged < 80 and ≥ 80 in the successful EVT group (DOCX 63 kb) [file 12883_2019_1237_MOESM6_ESM.docx]
