# Supplementary material for: Futile reperfusion and predicted therapeutic benefits after successful endovascular treatment according to initial stroke severity
Source: BMC Neurol. 2019 Jan 15;19:11. doi: 10.1186/s12883-019-1237-2 (PMC6332890; doi:10.1186/s12883-019-1237-2)
Supplement: Supplementary file 7 — Figure S4. The proportion of futile reperfusion according to each initial NIHSS category in the successful EVT group as a sensitivity analysis of EVT-treated patients within 6 h of onset. (DOCX 26 kb) [file 12883_2019_1237_MOESM7_ESM.docx]

Additional file 7: Figure S4 the proportion of futile reperfusion according to each initial NIHSS category in *the successful EVT group* as a sensitivity analysis of EVT-treated patients within 6 hours of onset.
