# Supplementary material for: Futile reperfusion and predicted therapeutic benefits after successful endovascular treatment according to initial stroke severity
Source: BMC Neurol. 2019 Jan 15;19:11. doi: 10.1186/s12883-019-1237-2 (PMC6332890; doi:10.1186/s12883-019-1237-2)

Additional file 8: Figure S5 The predicted therapeutic benefits of *the successful EVT* group (including only TICI grade 2b to 3) according to each of 4 initial NIHSS categories as a sensitivity analysis of EVT-treated patients within 6 hours of onset.


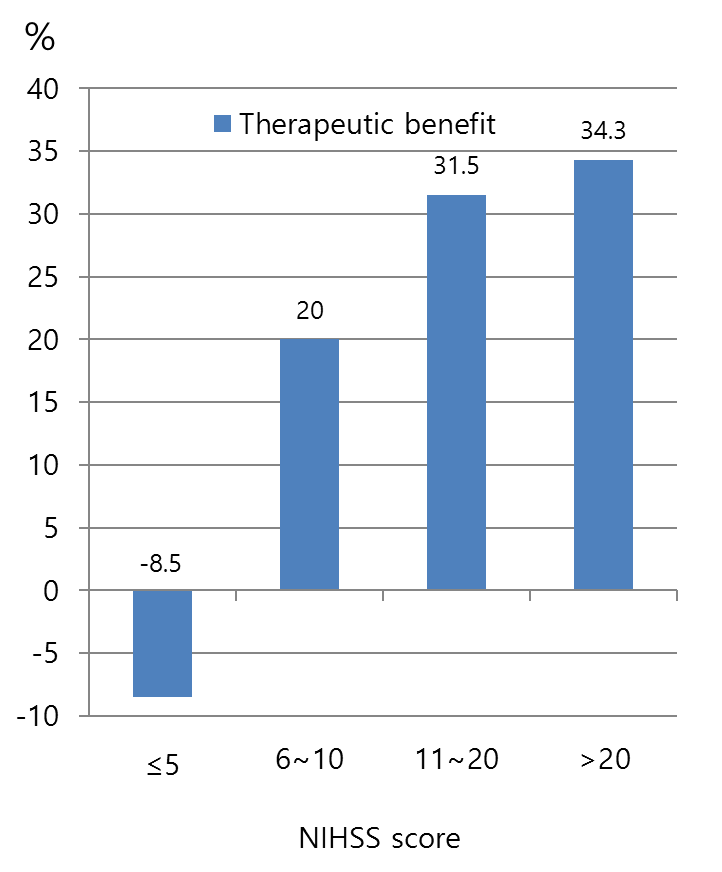

Supplement: Supplementary file 8 — Figure S5. The predicted therapeutic benefits of the successful EVT group (including only TICI grade 2b to 3) according to each of 4 initial NIHSS categories as a sensitivity analysis of EVT-treated patients within 6 h of onset. (DOCX 31 kb) [file 12883_2019_1237_MOESM8_ESM.docx]
