# Supplementary material for: Futile reperfusion and predicted therapeutic benefits after successful endovascular treatment according to initial stroke severity
Source: BMC Neurol. 2019 Jan 15;19:11. doi: 10.1186/s12883-019-1237-2 (PMC6332890; doi:10.1186/s12883-019-1237-2)
Supplement: Supplementary file 9 — Table S3. Proportions of EVT according to NIHSS category in acute ischemic stroke patients (n = 3117) who were treated with EVT or hospitalized within 12 h of onset and had causative ICA or MCA occlusion. (DOCX 15 kb) [file 12883_2019_1237_MOESM9_ESM.docx]

Additional file 9: Table S3. Proportions of EVT according to NIHSS category in acute ischemic stroke patients (n=3117) who were treated with EVT or hospitalized within 12 h of onset and had causative ICA or MCA occlusion

| Initial NIHSS Category | ≤5  (n=1247) | 6~10  (n=545) | 11~20  (n=1068) | >20  (n=257) | Total  (n=3117) |
| --- | --- | --- | --- | --- | --- |
| No-EVT | 94.4% (n=1177) | 75.2% (n=410) | 55.5%  (n=593) | 59.5%  (n=153) | 74.8%  (n.=2333) |
| Whole EVT | 5.6%  (n=70) | 24.8%  (n=135) | 44.5%  (n=475) | 40.5%  (n=104) | 25.2%  (n=784) |

EVT, endovascular treatment; NIHSS, National Institutes of Health Stroke Scale; ICA, internal carotid artery; MCA, middle cerebral artery
