# Supplementary material for: Futile reperfusion and predicted therapeutic benefits after successful endovascular treatment according to initial stroke severity
Source: BMC Neurol. 2019 Jan 15;19:11. doi: 10.1186/s12883-019-1237-2 (PMC6332890; doi:10.1186/s12883-019-1237-2)
Supplement: Supplementary file 10 — Table S4. Comparison of the Whole EVT group (regardless of reperfusion status) and the no-EVT group (DOCX 20 kb) [file 12883_2019_1237_MOESM10_ESM.docx]

Additional file 10: Table S4. Comparison of *the Whole EVT* *group* (regardless of reperfusion status) and *the no-EVT group*

|  | Whole EVT  n=784 | No-EVT  n=2333 | P-value |
| --- | --- | --- | --- |
| Age, mean±SD | 68.0±11.8 | 70.3±12.2 | 0.33‡ |
| Male, % | 444 (56.6) | 1319 (56.5) | 0.50* |
| TOAST |  |  | <0.001* |
| LAA | 179 (22.8) | 1074 (46.0) |  |
| CE | 430 (54.8) | 640 (27.4) |  |
| others | 175 (22.3) | 619 (26.5) |  |
| HTN, % | 493 (62.9) | 1521 (65.2) | 0.13* |
| DM, % | 171 (21.8) | 668 (28.6) | <0.001* |
| HL, % | 146 (18.6) | 429 (18.4) | 0.46* |
| Current Smoking, % | 172 (21.9) | 559 (24.0) | 0.13* |
| Atrial fibrillation, % | 411 (52.4) | 681 (29.2) | <0.001* |
| Onset to arrival§, minutes, IQR | 107 (50-212) | 292 (135-480) | <0.001† |
| NIHSS, IQR | 15 (10-18) | 5 (2-13) | <0.001† |
| NIHSS≤5 | 70 (8.9) | 1177 (50.5) | <0.001* |
| NIHSS 6~10 | 135 (17.2) | 410 (17.6) |  |
| NIHSS 11~20 | 475 (60.6) | 593 (25.4) |  |
| NIHSS >20 | 104 (13.3) | 153 (6.6) |  |
| Previous antithrombotics, % | 314 (40.1) | 855 (36.6) | 0.049* |
| Previous statin, % | 146 (18.6) | 438 (18.8) | 0.49* |
| SBP, mean±SD | 141.2±26.8 | 145.0±26.9 | 0.89‡ |
| Creatinine,mg/dL, mean±SD | 0.99±0.69 | 1.12±1.03 | <0.001‡ |
| Total cholesterol, mg/dL,mean±SD | 166.0±38.4 | 172.3±44.2 | <0.001‡ |
| Initial random glucose, mg/dL, mean±SD | 136.0±47.1 | 138.3±59.0 | 0.001‡ |
| Location of occlusion, % |  |  | <0.001* |
| MCA | 438 (55.9) | 1218 (52.2) |  |
| ICA | 196 (25.0) | 756 (32.4) |  |
| MCA+ICA | 150 (19.1) | 359 (15.4) |  |

EVT, endovascular treatment; LAA, indicates large artery atherosclerosis; CE, cardiac embolism; NIHSS, National Institutes of Health Stroke Scale; IQR, interquartile range; MCA, middle cerebral artery; ICA, internal carotid artery

* Calculated by Chi-squared test

† Calculated by Mann-Whitney U test

‡ Calculated by Student's t-test

§ Last known well time
