# Supplementary material for: Futile reperfusion and predicted therapeutic benefits after successful endovascular treatment according to initial stroke severity
Source: BMC Neurol. 2019 Jan 15;19:11. doi: 10.1186/s12883-019-1237-2 (PMC6332890; doi:10.1186/s12883-019-1237-2)
Supplement: Supplementary file 11 — Table S5. Distribution of stroke subtype by stratification of stroke severity in the Whole EVT group and no-EVT group. (DOCX 15 kb) [file 12883_2019_1237_MOESM11_ESM.docx]

Additional file 11: Table S5. Distribution of stroke subtype by stratification of stroke severity in the Whole EVT group and no-EVT group.

|  | LAA | CE | Others | P value |
| --- | --- | --- | --- | --- |
| NIHSS 0-5 | | | | <0.001 |
| Whole EVT | 28 (40.0) | 32 (45.7) | 10 (14.3) |  |
| no-EVT | 701 (59.6) | 161 (13.7) | 315 (26.8) |  |
| NIHSS 6-10 | | | | 0.03 |
| Whole EVT | 45 (33.3) | 60 (44.4) | 30 (22.2) |  |
| no-EVT | 177 (43.2) | 131 (32.0) | 102 (24.9) |  |
| NIHSS 11-20 | | | | <0.001 |
| Whole EVT | 90 (18.9) | 271 (57.1) | 114 (24.0) |  |
| no-EVT | 171 (28.8) | 254 (42.8) | 168 (28.3) |  |
| NIHSS >20 | | | | 0.90 |
| Whole EVT | 16 (15.4) | 67 (64.4) | 21 (20.2) |  |
| no-EVT | 25 (16.3) | 94 (61.4) | 34 (22.2) |  |

EVT, endovascular treatment; LAA, indicates large artery atherosclerosis; CE, cardiac embolism; NIHSS, National Institutes of Health Stroke Scale
